# Feasibility of concomitant cisplatin with hypofractionated radiotherapy for locally advanced head and neck squamous cell carcinoma

**DOI:** 10.1186/s12885-018-4893-5

**Published:** 2018-10-23

**Authors:** Alexandre Arthur Jacinto, Eronides Salustiano Batalha Filho, Luciano de Souza Viana, Pedro De Marchi, Renato de Castro Capuzzo, Ricardo Ribeiro Gama, Domingos Boldrini Junior, Carlos Roberto Santos, Gustavo Dix Junqueira Pinto, Josiane Mourão Dias, Heloisa Pelisser Canton, Raiany Carvalho, Lucas Augusto Radicchi, Soren Bentzen, Eduardo Zubizarreta, Andre Lopes Carvalho

**Affiliations:** 1 Department of Radiation Oncology, Barretos Cancer Hospital, Rua Antenor Duarte Villela, 1331, Barretos, SP 14.784-400 Brazil; 2 Department of Medical Oncology, Barretos Cancer Hospital, Rua Antenor Duarte Villela, 1331, Barretos, SP 14.784-400 Brazil; 3 Department of Head and Neck, Barretos Cancer Hospital, Rua Antenor Duarte Villela, 1331, Barretos, SP 14.784-400 Brazil; 4 International Atomic of Energy Agency - Vienna International Centre, PO Box 100, A-1400 Vienna, Austria; 5 University of Maryland School of Medicine, 655 W. Baltimore Street, Baltimore, MD 21201 USA

**Keywords:** Head and neck neoplasm, Accelerated radiation therapy, Hypofractionated radiotherapy, Chemoradiotherapy, Locally advanced head and neck carcinoma (LAHNC), Concomitant chemotherapy

## Abstract

**Background:**

The evolution of radiotherapy over recent decades has reintroduced the hypofractionation for many tumor sites with similar outcomes to those of conventional fractionated radiotherapy. The use of hypofractionation in locally advanced head and neck cancer (LAHNC) has been already used, however, its use has been restricted to only a few countries. The aim of this trial was to evaluate the safety and feasibility of moderate hypofractionated radiotherapy (HYP-RT) with concomitant cisplatin (CDDP).

**Methods:**

This single-arm trial was designed to evaluate the safety and feasibility of HYP-RT with concomitant CDDP in LAHNC. Stage III and IV patients withnonmetastatic disease were enrolled. Patients were submitted to intensity modulatedradiation therapy, which comprised 55 Gy/20 fractions to the gross tumor and44–48 Gy/20 fractions to the areas of subclinical disease. Concomitant CDDPconsisted of 4 weekly cycles of 35 mg/m2. The primary endpoints were the treatment completion rate and acute toxicity.

**Results:**

Twenty patients were enrolled from January 2015 to September 2016, and 12 (60%) were classified as unresectable. All patients completed the total dose of radiotherapy, and 19 patients (95%) received at least 3 of 4 cycles of chemotherapy. The median overall treatment time was 29 days (27–34). Grade 4 toxicity was reported twice (1 fatigue and 1 lymphopenia). The rates of grade 3 dermatitis and mucositis were 30% and 40%, respectively, with spontaneous resolution. Nasogastric tubes were offered to 15 patients (75%) during treatment; 4 patients (20%) needed feeding tubes after 2 months, and only 1 patient needed a feeding tube after 12 months.

**Conclusion:**

HYP-RT with concomitant CDDP was considered feasible for LAHNC, and the rate of acute toxicity was comparable to that of standard concomitant chemoradiation. A feeding tube was necessary for most patients during treatment. Further investigation of this strategy is warranted.

**Trial registration:**

ClinicalTrials, NCT03194061. Registered 21 Jun 2017 – Retrospectively registered.

## Background

Concomitant chemoradiotherapy (cCRT) improves loco-regional control (LRC) and overall survival (OS) in locally advanced head and neck cancer (LAHNC) compared with radiotherapy (RT) alone; consequently, chemoradiation is the standard of care for these patients [1]. Three-week 100 mg/m^2^ cisplatin concomitant with conventional fractionation radiotherapy (CFRT - 35 2-Gy fractions over 7 weeks) is the most studied regimen and is associated with significant toxicity, which compromises patient compliance and may not be suitable for all patients [2–5].

Altered fractionation is an alternative for patients who are not suitable for cCRT and can improve OS compared with CFRT alone [6, 7]. Accelerated RT, in which the total dose is delivered in a short period of time, has radiobiological advantages and is also associated with improved clinical outcomes [8, 9]. Hypofractionation is an attractive method for accelerating RT and has been used with success with other tumor sites, showing comparable outcomes and a reduced cost compared to those of CFRT [10–13]. A remarkable moderate hypofractionated RT (HYP-RT) schedule for head and neck cancer, which delivers 55 Gy in 20 fractions (2.75 Gy per fraction) for 5 days per week, has been described in Birmingham/Edinburgh [14]. The biologically effective dose (BED) of the HYP-RT is approximately the same of CFRT [15]. The United Kingdom Head and Neck (UKHAN1) trial was one of the largest trial to demonstrate the superiority of cCRT over RT alone for LAHNC. In the UKHAN1 trial, almost 50% of patients were submitted to hypofractionated RT, including the HYP-RT schedule, and hypofractionation did not affect event-free survival compared with CFRT. The chemotherapy regimen used in the UKHAN1 trial was non-platin-based and, to the best of our knowledge, no data exists regarding HYP-RT concomitant with CDDP [16].

Patients from low- and middle-income countries (LMIC) have limited resources for RT and face long waiting times to be treated [17, 18]. Consequently, in addition to the radiobiological and clinical benefits of accelerated RT, hypofractionation regimes can also be an important strategy to shorten treatment times and thus improve access to RT. Additionally, a short RT schedule is associated with better patient compliance [19].

The aim of this trial was to evaluate the feasibility and early safety of concomitant cisplatin in combination with HYP-RT in a high-volume center from an LMIC.

## Methods

### Patients

This open-label trial enrolled patients according to the following eligibility criteria: 1) biopsy-proven, non-metastatic, squamous cell carcinoma of the oropharynx, larynx and hypopharynx; 2) advanced stage disease, namely, stage III, IVa or IVb; 3) an ECOG performance status between 0 and 2; 4) aged from 18 to 70 years old; and 5) no history of previous malignancy. Each case was extensively discussed in multidisciplinary head and neck tumor board (HNTB) meetings to define the stage, resectability status and treatment recommendation. The HNTB comprised radiation oncologists, medical oncologists, radiologists and head and neck surgeons.

The study protocol was approved by the institutional review board and was conducted according to the Helsinki declaration. All patients provided specific written informed consent prior to participating in this trial. The study was also registered at ClinicalTrials.gov under the number NCT03194061.

### Treatment

#### Radiation therapy

All patients were immobilized with a 5-point head-shoulder thermoplastic mask and subjected to a computed tomography (CT) simulation with intravenous contrast. The primary tumor and gross nodal tumor were defined as the gross target volume (GTV). The clinical target volume of high risk (CTVhi) was an expansion of 0.5 cm from the GTV. Nodal neck delineation was based on a multi-institutional consensus guideline [20]. All regions deemed to be at high risk of subclinical disease were delineated by the physician as intermediate risk and named CTVInt. Nodal regions with a low risk of harboring subclinical disease were defined as CTVlow. Another tridimensional 0.5-cm expansion from each CTVs was performed to generate the respective planning target volume (PTV). The simultaneous integrated boost (SIB) intensity modulated radiation therapy (IMRT) technique was used for all cases once per day for 5 days per week. The total dose for PTVhi was 5500 cGy in 20 fractions of 275 cGy (RT 55 Gy). Assuming the α/β ratio to be 10Gy for head and neck carcinoma, the estimated BED for HYP-RT is about 66Gy_10,_ which is almost equivalent to CFRT [15]. Areas of intermediate risk (PTVint) for subclinical disease received 4800 cGy, and areas of lower risk for subclinical disease (PTVlow) received 4400 cGy. To optimize the overall length of treatment, all radiotherapy started on a Monday. The treatment plans were normalized to deliver 100% of the prescribed dose to at least 95% of each PTV (D_95%_ = 100%). Additionally, no more than 10% of the PTVhi received a dose higher than 60.5 Gy (V_60.5Gy_ < 10%), and no more than 1% of the PTV received < 90% of the dose (D_99%_ > 90%).

#### Chemotherapy

Patients received intravenous cisplatin at a dose of 35 mg per square meter on days 1, 8, 15 and 22, concomitant with radiotherapy. Pre-medications were performed according to the institutional protocol. No dose reduction was allowed. Chemotherapy was delayed up to 2 weeks in cases of grade 3 or 4 toxicity. Subsequent administration was allowed if toxicity declined to below grade 2.

### Assessments

A baseline assessment was performed with a medical history, physical examination, flexible video laryngoscopy, a video deglutogram, and CT scan of the head, neck and chest. Human papillomavirus positivity was tested in all oropharyngeal cases. p16 immunohistochemistry was performed on formalin-fixed paraffin-embedded tissue sections that were cut to 4-μm thicknesses using the CINtec p16INK4A assay, according to the manufacturer’s instructions (CINtec Histology Kit; Ventana Medical Systems, Tucson, AZ, USA).

All patients were evaluated and graded for evidence of developing treatment toxicity according to the National Cancer Institute-Common Terminology Criteria for Adverse Events (NCI-CTCAE) version 4.0, except for radiation mucositis, which was graded according to NCI-CTCAE version 3.0. Assessments of patient adverse events were performed weekly during and up to 1 month after treatment, every month up to 4 months and then every 3 months up to 2 years.

All responses were defined by the HNTB. Tumor response evaluations were performed after 8 weeks and 16 weeks of treatment. The response assessment was performed by physical examination, head & neck CT and video laryngoscopy. Response was based on the Response Evaluation Criteria in Solid Tumors (RECIST), version 1.1. [^18^F] fluorodeoxyglucose positron emission tomography/CT (FDG-PET/CT) was not available for all patients during the response evaluation.

### Support therapy

A dedicated oncology dietician evaluated all patients at baseline, weekly during and up to 1 month after treatment, and every 1–3 months thereafter according to the patient needs. During each visit, the anthropometric parameters were collected; the body mass index (BMI) and percent weight loss were calculated, and dietary intake was estimated by 24-h recall. All patients received oral glutamine during RT, and nutritional supplementation was promptly initiated for patients with minimal weight loss. Patients with weight loss higher than 5–10% of the initial body weight (defined as the weight measured before treatment) and patients with grade 3 dysphagia were offered nasogastric tubes (NGTs).

Prophylactic and therapeutic photobiomodulation (PBM) with a low-level laser for oral mucositis was offered to all patients during treatment. All patients had post-diagnosis follow-up visits with a speech therapist.

### Study design and statistical analysis

The primary endpoint of the study was the completion rate of treatment. The treatment was considered completed for patients who received at least 90% of the radiation dose (49.5 Gy), with a cumulative cisplatin dose of 105 mg/m^2^ (at least 3 cycles) and with an overall treatment time (OTT) below 35 consecutive days.

Toxicity was another primary endpoint, and we determined that the treatment would be considered unsafe if the rate of grade 4 mucositis and dermatitis was higher than 15%. No data exists on the use of concomitant CDDP concurrent with HYP-RT. In our previous investigation, the rate of patients who received at least 2 of 3 cycles of cCRT after induction chemotherapy was approximately 85% [21]. We expected that at least 75% of patients would complete the treatment with no significant delay and no severe acute toxicity. Therefore, we could conclude the feasibility of this protocol with 95% certainty if 15 of 20 patients completed the treatment as stated.

### Stopping rule

The safety analysis of the HYP-RT and concurrent chemotherapy was performed in a three-steps process within the study. To evaluate the completion rate and acute toxicity, we defined that each patient had to have a minimal follow up of 3 months after the cCRT:

A) STEP 1: In the first step, 5 unresectable cases were included. The criteria for moving forward to STEP 2 was that at least 3 patients (3/5–60%) could complete the treatment as described in Section “Study design and statistical analysis”. If 3 or more patients had not completed the treatment then the study protocol would be deemed too toxic and the study would be re-evaluated with respect to the feasibility.
B) STEP 2: Five more patients - resectable or unresectable – were included. The criteria for moving to STEP 3 was that at least 7 patients (7/10–70%) could complete the treatment as described in Section “Study design and statistical analysis”. If 4 or more patients (among the first 10 patients) had not completed the treatment then the study protocol would be deemed too toxic and the study would be re-evaluated with respect to the feasibility.
C) STEP 3: Finally, after the confirmation of the protocol safety among the first 10 patients, the STEP 3 was commenced including 10 more patients up to a total of 20 cases. The study protocol would be considered feasible if at least 15 patients (15/20–75%) could complete the treatment as described in Section “Study design and statistical analysis”. If 6 or more patients had not completed the treatment then the study protocol would be deemed too toxic and not feasible.

Additionally, a dose-limiting toxicity (DLT – defined as any grade 4 toxicity) was established. Basead on standard cCRT arm of RTOG 0522, RTOG 9501 and our previous experience on LAHNC, the DLT estimated for this study was 25%. After each STEP, If the rate of toxicity exceeded the estimated DLT or if at any time a Grade 5 (death) was registered, the accrual would be stopped and the protocol had to be evaluated by an independent data monitoring committee with respect to the feasibility of the study protocol.

## Results

### Patient characteristics

**Fig. 1:**
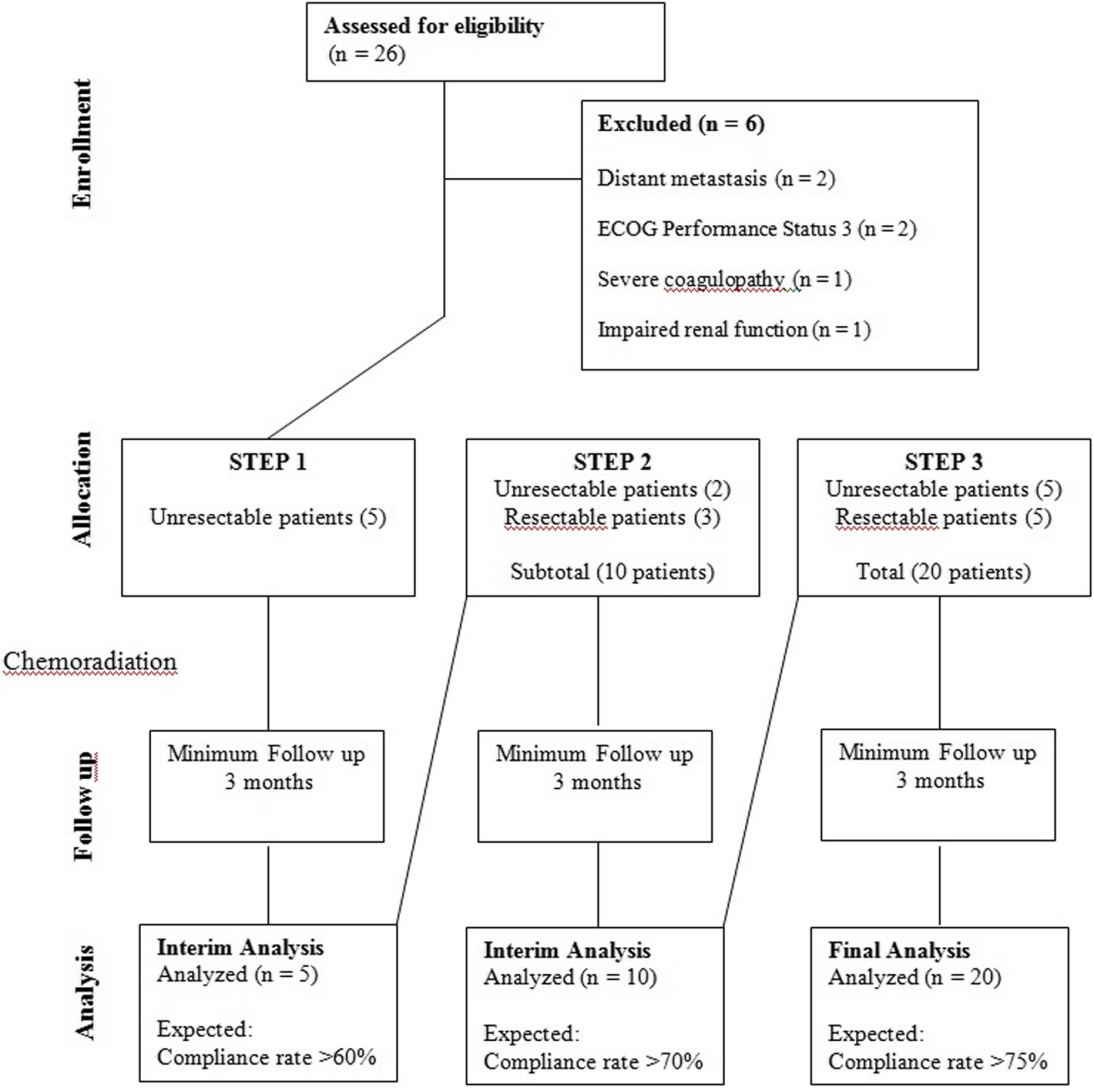
CONSORT diagram showing the flow of participants through each steps of the feasibility trial

From January 2015 to September 2016, 20 patients were enrolled in this study. The accrual rate was mainly determined by the protocol stopping rule. Figure 1 presents a CONSORT diagram to demonstrate the patient flow in the protocol. The patient characteristics are listed on Table 1. The median age was 53-years (42–69), 16/20 (80%) were male and 19/20 (95%) had a history of smoking. Most patients had stage IV disease (75%), T3/T4 (80%) and N2/N3 (75%). The disease was considered unresectable in 12/20 patients (60%), and there were no HPV-related tumors.

**Table 1:** Patient characteristics

Median age–years (range)	53 (42–69)
	Patients (%)
Gender	
Male	16 (80)
Female	4 (20)
Primary site	
Oropharynx	10 (50)
Larynx	6 (30)
Hypopharynx	4 (20)
Performance status (ECOG)	
0	4 (20)
1	14 (70)
2	2 (10)
Clinical stage	
III	5 (25)
IV	15 (75)
Tumor (T) - Stage	
T1	1 (05)
T2	3 (15)
T3	8 (40)
T4	8 (40)
Nodal (N) - Stage	
N0	5 (25)
N1	0 (00)
N2	11 (55)
N3	4 (20)
Resectability	
Resectable	8 (40)
Unresectable	12 (60)
Smoking status	
Current	11
Former	8
Never	1

### Treatment compliance

Treatments started up to 10 days after accrual, and all patients were treated per protocol. In the STEP 1 it all patients completed the treatment according to the protocol (Section “Study design and statistical analysis”), and the protocol was moved forward to the next STEP. In STEP 2, 70% of completion was expected and 90% of patients completed the treatment. In STEP 3 It was expected that at least 75% of all 20 patients had completed cCRT. Nineteen of 20 patients (95%) received at least 3 cycles of chemotherapy. All patients received the total dose of RT. The median treatment time was 29 days (27–34) and the length of treatment was greater than 30 days in 1 patient. The completion rate was 95% then study protocol was considered feasible. The number of patients who received 4, 3 and 2 chemotherapy cycles was 11, 8 and 1, respectively. Table 2 outlines the completion rate according to each step of the protocol.

**Table 2:** Feasibility criteria and completion rate according to each step of the trial. The results are presented according to each feasibility criterion (per treatment)

	Target Range	Delivered Median (range)
STEP 01 (5 unresectable patients)		
Radiotherapy dose	49.5–55 Gy	55 Gy (55)
Cisplatin dosage (mg/m^2^)	105–140	140 (105–140)
Overall treatment time (days)	< 35	29 (29–30)
STEP 02 (step 01 + 5 patients) Resectable or unresectable		
Radiotherapy dose	50–55 Gy	55 (55)
Cisplatin dosage (mg/m^2^)	105–140	140 (70–140)
Overall treatment time (days)	< 35	29 (27–34)
STEP 03 (step 2 + 10 patients) Resectable or unresectable		
Radiotherapy dose	50–55 Gy	55 (55)
Cisplatin dosage (mg/m^2^)	105–140	122 (70–140)
Overall treatment time (days)	< 35	29 (27–34)

### Toxicity

No treatment-related deaths occurred. Only 2 patients (10%) experienced grade 4 toxicity (fatigue and lymphopenia). No patient presented grade 3 neutropenia. The rate of grade 3/4 lymphopenia was 20%. Renal function was stable, and only 1 patient (5%) showed a small creatinine elevation (grade 1) during treatment. Table 3 presents the most important toxicities observed during treatment.

**Table 3:** Acute toxicity of concurrent cisplatin with hypofractionation (HYP-UK) for locally advanced head and neck cancer. According to the Common Terminology Criteria for Adverse Events Version 3.0

Acute adverse event	Grade 1/2	Grade 3	Grade 4
	n (%)	n (%)	n (%)
Hematological			
Anemia	7 (35)	0	0
Lymphopenia	7 (35)	3 (15)	1 (5)
Thrombocytopenia	1 (5)	0	0
Neutropenia	5 (25)	1 (5)	0
Febrile Neutropenia	0	1(5)	0
Non-Hematological			
Radiodermatitis	19 (95)	6 (30)	0
Mucositis	15 (75)	8 (40)	0
Increased serum creatinine	1 (5)	0	0

**Fig. 2:**
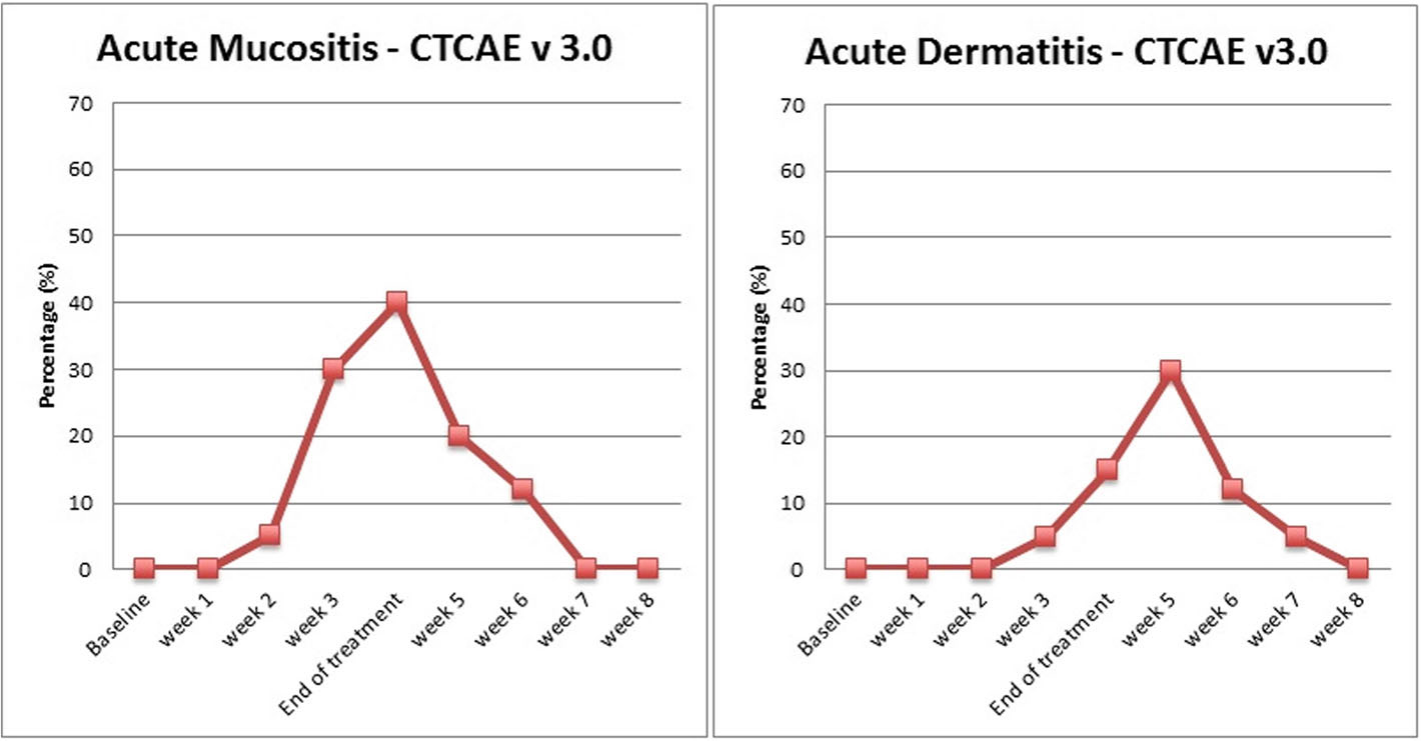
Rate of mucosistis and radiation dermatitis from the treatment start (baseline). According to the Common Terminology Criteria for Adverse Event version 3.0

No grade 4 dermatitis or mucositis occurred. The rate of grade 3 dermatitis was 30%, and dermatitis was more frequent during the first week after treatment. Eight patients (40%) experienced grade 3 mucositis during the last week of treatment. As outlined in Fig. 2, all patients had complete resolution of the mucositis and dermatitis up to 1 month after treatment.

Most patients (95%) lost weight during treatment, and the median percentage of weight loss during treatment was 7.8%. All patients received nutritional supplementation during treatment, and 15 patients (75%) required NGT; most patients required NGTs due to weight loss > 5% and grade 3 dysphagia or odynophagia. At the last follow-up visit, only one patient was still using the NGT.

### Tumor response and salvage surgery

The overall rate of response (T and N) was 95% after 2 months. The rate of complete response (CR) of the primary site (T) was 85%. The nodal CR was 40%. The overall CR was 85% for patients with resectable disease and 35% for patients deemed unresectable.

## Discussion

Altered fractionation is a well-established alternative of RT in the LAHNC treatment because many studies have demonstrated its superiority in disease control and survival compared with CFRT [7]. By reducing the OTT, the accelerated repopulation effect is minimized, which may explain the improved outcomes when treatment is accelerated [8, 9, 15]. Hypofractionation is a remarkable method for accelerating cancer treatment and is associated with better RT compliance [19]. Additionally, radiobiological and long-term clinical data have suggested that the HYP-RT regimen of 55 Gy in 20 fractions is, at least, equivalent to CFRT for LAHNC [15, 16]. However, despite recent technological RT advances and successes in other tumor sites [10–12], the use of hypofractionation regimens with radical intent in LAHNC is modest and restricted to a few countries, particularly the United Kingdom [14, 16, 22–24]. The main reason for this restriction is the toxicity concern regarding the high dose per fraction, notably with concomitant chemotherapy [25]. Moreover, whether concomitant CDDP improve outcomes in the context of hypofractionation for LAHNC is unknown. The long-term outcomes of the UKHAN1 trial, which included CFRT and HYP-RT, demonstrated good compliance, a low rate of late toxicity, improved disease control, fewer new tumors and reduced mortality when cCRT was compared to RT alone [16]. Nevertheless, the chemotherapy used in the UKHAN1 trial was non-platin based. Although Madhava and colleagues have already demonstrated the feasibility of carboplatin with HYP-RT, to the best of our knowledge, our trial is the first to address the feasibility of concurrent CDDP with hypofractionation in LAHNC [14]. With a 95% of completion rate, our early data demonstrate the good compliance and suggest the feasibility of this protocol for patients from a middle-income country.

The standard concomitant chemotherapy with CFRT comprises the full dose of CDDP (3 cycles of 100 mg/m^2^ every 21 days), and the treatment is associated some toxicity, poor treatment compliance and treatment delays [2, 4, 5]. Indeed, standard cCRT may not be suitable for all patients [1, 3]. In a Brazilian report of CRT for patients with unresectable, stage IV, non-metastatic disease, the full dose of CDDP was not feasible due to high treatment-related toxicity and unplanned treatment breaks [3]. Although we were concerned about severe toxicity, two patients only (10%) experienced grade 4 toxicity during treatment (fatigue and lymphopenia) and the established DLT was not reached. In recent trials which the control arms was the standard cCRT, most IMRT based, the rate of grade 3–4 mucosistis, disfagia and neutropenia was 38–40%, 32–70%, and 19–26%, respectively [26–28]. Comparing with those IMRT based trials our rate of grade 3 neutropenia, mucositis and dermatitis was considered acceptable [26–28]. The standard cCRT in the control arm of RTOG 0129 was associated with high rate of enteral tube need (70%) during treatment and the long term need was 36% [26]. In the current trial NGT was offered to 75% of patients during treatment, but 4 patients (20%) and 1 patient only (5%) were still using NGT 1 month and 12 months after treatment, respectively.

The most important radiobiological advantage of hypofractionation involves its ability to enable explorations aimed towards reducing the OTT and, thereafter, minimizing the effect of accelerated repopulation of head and neck squamous cell carcinoma [15]. A median local control reduction of 1.4% per day of GAP and approximately 11% per week of OTT prolongation have been estimated [29, 30]. LAHNC tumor burden and treatment toxicity are associated with significant suffering and disability and are the primary causes of treatment interruptions [31]. Because this was a safety trial, we determined that the OTT was as important as the completion rate, which used the OTT as a parameter to define treatment feasibility. Indeed, we were concerned that by increasing acute toxicity, the OTT would be prolonged due to unplanned treatment breaks. However our data suggest the good tolerance profile of the protocol, whereby no patient needed a treatment break due to toxicity and no significant delay was experienced. In addition to treatment toxicity, many other factors underlie RT prolongation, including low socioeconomic status, a long treatment course, an unplanned equipment breakdown and the travel distance from the patient’s home to the RT site [19]. An integrated multidisciplinary approach plays an essential role in improving tolerance and RT compliance in LAHNC [31, 32]. In our institution, all head and neck patients receive continuous assistance of a social worker, and they are followed by a well-structured supportive care team during treatment and follow-up. All patients in the protocol were offered laser therapy for the normal mucosa, oral glutamine, early nutritional interventions and speech therapy during and after treatment. We believe that our integrated multidisciplinary care had a positive impact on treatment compliance, and a limitation of our outcomes may be that they are not representative of many centers from LMIC which cannot provide good integrated supportive care for patients with LAHNC OS and disease-free survival are the main endpoints for assessing the effectiveness of conservative treatment for LAHNC; however, the tumor response rate may be a surrogate of long-term LRC and survival, although this has not been reported in most prospective trials [33]. Despite the advanced tumor stages in our population the overall tumor response in our trial was consistent with the literature and with our previous experience with LAHNC [21].

According to the International Atomic Energy Agency (IAEA), an estimated 50% or more of RT patients will not have access to treatment in LMIC. Again, recent improvements in RT delivery have allowed the safe use of hypofractionation in many other cancer sites, and short-course RT is associated with cost effectiveness, patient convenience and better compliance [10–12, 17–19]. Thus, HYP-RT for LAHNC may be an important approach towards improving RT resource sparing. The design, short follow-up time and small patient number of this single institutional feasibility trial does not allow a strong conclusion about long-term outcomes; however, these findings must be further explored in large prospective clinical trials.

Currently, the IAEA is conducting a phase 3 trial, known as the HYPNO-trial (NCT02765503), to compare hypofractionation with accelerated normo-fractionated RT for LAHNC. Another prospective trial from the University of Birmingham, the COMPARE trial (EudraCT No: 2014–003389-26), is now comparing standard cCRT with hypofractionated RT with concomitant CDDP. Both studies are currently active and open for recruitment.

## Conclusion

In summary, treatment of LAHNC with HYP-RT concurrent with cisplatin appears feasible and safe and is associated with a good response rate. These data highlight the potential usefulness of hypofractionation for LAHNC, especially for LMIC, where access to RT is poor. Long-term outcome data from the HYPNO and COMPARE trials are expected to provide definitive conclusions about HYP-RT for LAHNC.
